# Antibiotic versus surgery in the treatment of acute appendicitis in the pregnant population: A systematic review and meta-analysis

**DOI:** 10.12688/f1000research.129906.1

**Published:** 2023-02-16

**Authors:** Valeska Candrawinata, Ricarhdo Hanafi, Bernard Agung Baskoro, Andry Irawan, Christofani Ekapatria, Natalia Maria Christina, Heru Sutanto Koerniawan, Freda Halim

**Affiliations:** 1 Faculty of Medicine, Pelita Harapan University, Tangerang, Banten, 15811, Indonesia; 2 Division of Oncology, Department of Surgery, Faculty of Medicine, Pelita Harapan University, Siloam General Hospital, Tangerang, Banten, 15811, Indonesia; 3 Division of Digestive Surgery, Department of Surgery, Faculty of Medicine, Pelita Harapan University, Siloam General Hospital, Tangerang, Banten, 15811, Indonesia; 4 Division of Reproductive Endocrinology and Fertility, Department of Obstetrics and Gynecology, Faculty of Medicine, Pelita Harapan University, Siloam General Hospital, Tangerang, Banten, 15811, Indonesia; 5 Department of Surgery, Faculty of Medicine, Pelita Harapan University, Siloam General Hospital, Tangerang, Banten, 15811, Indonesia; 6 Department of Surgery, Pelita Harapan University, Tangerang, Banten, 15811, Indonesia

**Keywords:** Antibiotic, Surgery, Acute Appendicitis, Pregnant

## Abstract

**Introduction:** Acute appendicitis is the most common surgical emergency in pregnant women. There has been a wide variance in clinical practice worldwide, with some favoring an antibiotic-only approach while others prefer surgery as the first-line management. Therefore, we designed the current analysis to synthesize the available evidence on the efficacy and safety of antibiotics versus surgery management.

**Methods:** We searched PubMed, Scopus, EuropePMC, and Cochrane Central from March 4, 1904 until November 25, 2022, to look for studies comparing antibiotics and surgery in pregnant patients with acute appendicitis. We only included studies that provided a comparison between the two treatments. We included preterm delivery, fetal loss, maternal death, and complications as outcomes. The results were compared using an odds ratio and 95% confidence interval. We also performed a sensitivity analysis by excluding studies with a serious risk of bias.

**Results:** We included five non-randomized studies for the analysis. We found that patients in the antibiotic group had a lower risk of preterm labor (OR 0.63 [95% CI 0.43–0.92]; p 0.02) but a higher risk of complications (OR 1.79 [95% CI 1.19–2.69]; p 0.005). We did not find any difference in the other outcomes.

**Conclusion:** The increased risk of complications should caution clinicians about using antibiotics as the first-line management. More studies are required to identify patients who would benefit the most before antibiotics could be adopted as a treatment for acute appendicitis in pregnant patients.

## Introduction

The prevalence of acute appendicitis in pregnancy is quite high, making it one of the most common surgical emergencies in the pregnant population. Its prevalence ranges from 1 in 700 to 1500 live births. ^1^^–^^3^ Although not as common as other non-surgical emergencies, acute appendicitis has dire consequences both for the mother and her fetus. Acute appendicitis correlates with placental abruption, preterm labor, fetal loss, and even maternal death.

Acute appendicitis in pregnancy could be treated with surgery, either open or laparoscopic appendectomy, or with antibiotics alone. The 2020 update of the World Society of Emergency Surgery (WSES) Jerusalem guidelines gave a low-strength recommendation against treating acute appendicitis non-operatively during pregnancy and recommended using laparoscopic appendectomy. ^4^ One of the reasons for this recommendation was the lack of high-level evidence, where they only considered three studies, of which two were case reports. As a result, clinical practices regarding the treatment of acute appendicitis in pregnancy worldwide continued to vary from one region to another. In Korea, 25% of pregnancy affected by acute appendicitis was treated conservatively, ^5^ compared to 63% in China, ^6^ 67% in Japan, ^7^ and 45% in the United States of America. ^8^ This wide variance and high usage of conservative strategy could mean that not all clinicians adopted the existing guideline.

Some considerations for treating acute appendicitis conservatively were the lack of surgical resources, patient’s preference, and uncomplicated type of appendicitis. There has also been controversy regarding the type of appendectomy, with one systematic review reporting a higher proportion of fetal loss in patients treated with laparoscopic appendectomy. ^9^ Moreover, two meta-analyses have reported that antibiotics treatment alone for uncomplicated appendicitis in the general population had a lower rate of complications, with an acceptable, albeit lower, rate of success. ^10^^,^^11^ Therefore, seeing the lack of strong evidence and variance in clinical practice, we planned the current review to identify the efficacy and safety of conservative treatment in the treatment of acute appendicitis in pregnancy.

## Methods

### Database and literature search

We searched PubMed, Scopus, EuropePMC, and Cochrane Central from the March 4, 1904 until November 25, 2022, using the following keywords: [“acute appendicitis” or “appendicitis” and “pregnancy” and “conservative” or “antibiotic” or “antibiotics”]. We also reviewed the bibliographies of relevant studies to identify any other studies. We only included English-language literature. The initial search and screening were done by two independent authors. Any discrepancies were resolved by discussion with a third author.

### Study selection/design

The inclusion criteria were: 1) types of patients: pregnant patients with uncomplicated and complicated acute appendicitis; 2) types of intervention: conservative management with antibiotics alone; 3) types of comparison: open or laparoscopic appendectomy; 4) analyzed outcomes: preterm delivery, fetal loss, maternal death, and complications; and 5) types of studies: prospective and retrospective studies. We defined complications as those arising from the pathological processes or treatment, including, but not limited to, sepsis or septic shock, pneumonia, venous thromboembolism (VTE), and surgical site infection. Studies that did not provide the aforementioned variables as outcomes were excluded. We excluded review articles, editorials, case reports, and case series. This analysis was started in July 31, 2022 and finished in November 9, 2022.

### Data extraction

After the studies were selected, two authors independently extracted the data on study authors, publication year, study design, patients’ characteristics, treatment characteristics, and outcomes using a standardized form. Patients were then dichotomized according to the treatment they received.

### Statistical analysis

We inputted patients’ data in a 2-by-2 contingency table according to the analyzed outcomes. We compared each outcome between conservative and surgical groups using odds ratios (OR) and 95% confidence intervals (CI). Heterogeneity among studies was evaluated using the *I ^2^* statistic and Cochrane *Q-*statistic test. An *I ^2^* value higher than 40% or a P value higher than 0.10 indicated a significant presence of heterogeneity. However, a random-effect model was used regardless of heterogeneity because we expected the studies to have significant differences in the choice of antibiotics. We also planned a subgroup analysis according to whether the studies’ participants had uncomplicated or complicated appendicitis. As performing a randomized controlled trial on a pregnant population is ethically difficult, we expected most studies to be non-randomized. Therefore, we also planned a sensitivity analysis by excluding studies with a serious risk of bias.

We assessed the risk of bias using the Cochrane risk-of-bias tool (The Cochrane Collaboration) for randomized studies and the Risk of Bias in Non-randomized Studies - of Intervention tool (ROBINS-I) for non-randomized studies. We used a funnel plot analysis to assess publication bias. We graded the strength of evidence for each outcome using the Grading of Recommendations Assessment, Development, and Evaluation (GRADE) approach. We used the Review Manager version 5.4 (The Cochrane Collaboration) ( https://training.cochrane.org/online-learning/core-software/revman/revman-5-download) program for all statistical analysis. We conducted this study according to the 2015 PRISMA guidelines for a systematic review. This review has been registered at The International Prospective Register of Systematic Reviews (PROSPERO) under the registration number CRD42022371119.

## Results

**Figure 1.**
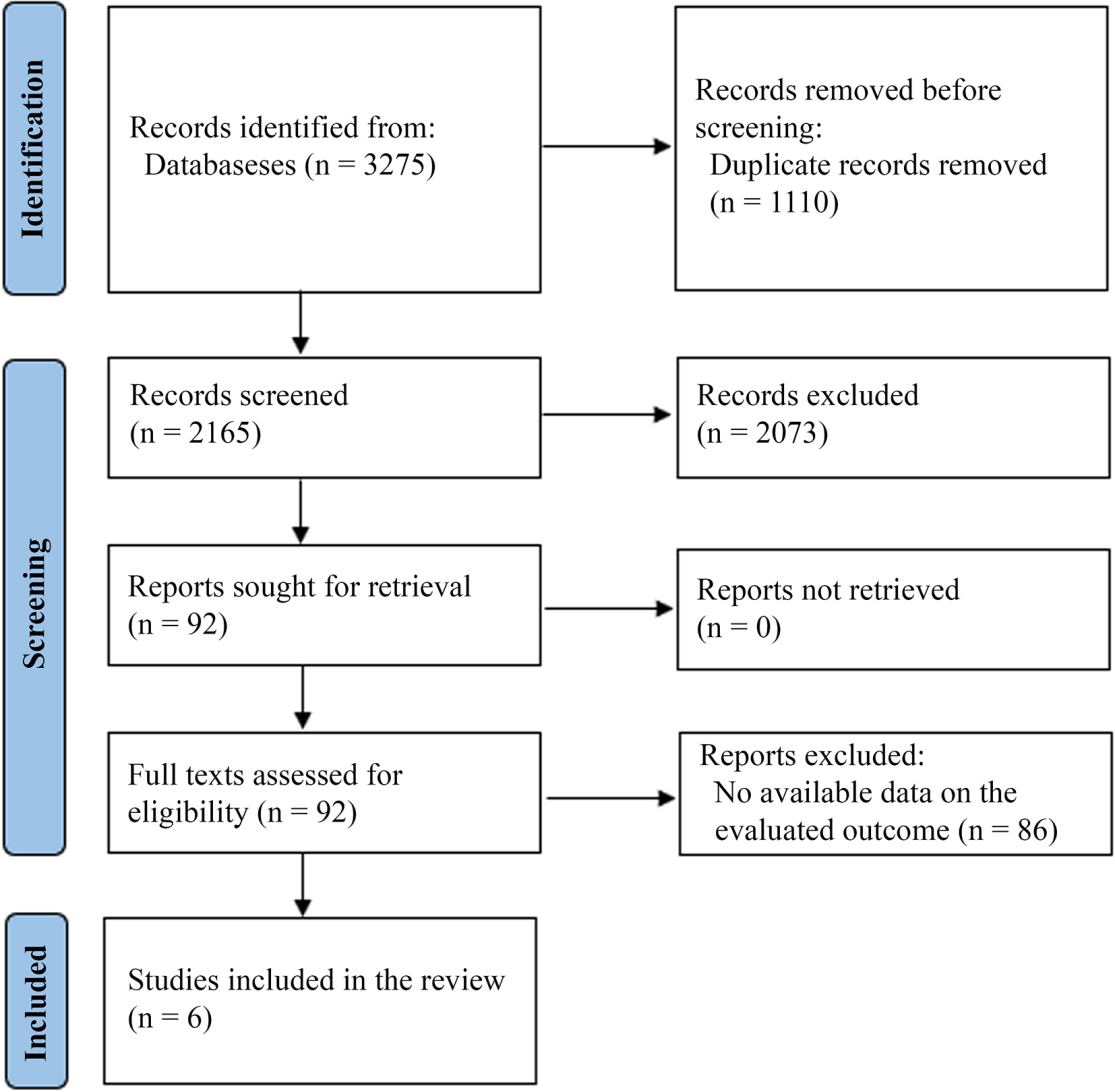
PRISMA flow diagram.

Our initial search resulted in 3,275 records. After duplicate removal, 2,165 titles and abstracts were screened. This initial screening resulted in 92 studies being sought for retrieval. All studies were retrieved, and we assessed their full texts for eligibility. We excluded 87 studies because they did not provide a comparison between antibiotics and surgery and did not provide data on the outcomes we planned to analyze. Finally, five studies were included for final analysis ( Figure 1). ^6^^,^^7^^,^^12^^–^^14^

### Study characteristics

The characteristics of the included studies are presented in Table 1. All of the studies were non-randomized and mostly retrospective. Patients were mostly 25 to 35 years old and in their second trimester. Liu *et al*. included only uncomplicated appendicitis case ^6^ while Abbasi *et al*., Nakashima *et al.*, and Vasileiou *et al*. included both complicated and uncomplicated appendicitis case. ^7^^,^^13^ However, Yang *et al*. did not report the severity of appendicitis in their samples. ^14^ Four studies reported their antibiotic use. ^6^^,^^7^^,^^12^^,^^14^ Third-generation cephalosporin was the main choice. Five studies reported surgery type, and all used either open or laparoscopic appendectomy. ^6^^,^^7^^,^^12^^,^^14^

**Table 1.** Characteristics of included studies.

Authors & year	Study design	Cohort Size (n)	Age (yrs)	Trimester	Complicated	Antibiotics	Surgery	Outcome
Abbasi *et al.*, 2014 ^1^	Retrospective cohort	413/6701	<25: 42.5%; 25–25: 48.5%; >35: 9.0%	Not specified	7271 (20.3%)	Not specified	Open and laparoscopic	SIRS, sepsis, or severe sepsis; septic shock; peritonitis; VTE
Liu *et al.*, 2021 ^6^	Retrospective cohort	34/20	29.0 ± 4.1 / 30.5 ± 4.8	1 ^st^ trimester: 8 (23.5%) / 8 (40%); 2 ^nd^ trimester: 20 (58.8%) / 9 (45%); 3 ^rd^ trimester 6 (17.6%) / 3 (15%)	All uncomplicated	Ceftriaxone, ertapenem/meropenem, ceftriaxone + metronidazole	Open and laparoscopic	Preterm delivery; mode of delivery; fetal loss; birth weight; APGAR scores; postpartum complications; maternal death; LOS; recurrence.
Nakashima *et al.*, 2021 ^7^	Retrospective cohort	113/56	29.7 ± 5.6 / 30.1 ± 4.2	1 ^st^ trimester: 26 (23%) / 12 (21%); 2 ^nd^ trimester: 54 (48%) / 33 (59%); 3 ^rd^ trimester 31 (27%) / 9 (16%); unknown: 2 (2%) / 2 (4%)	7 (6%) / 23 (41%) ^*^	2 ^nd^, 3 ^rd^, 4 ^th^ generation cephalosporin	Open and laparoscopic	Fetal loss; maternal death; SSI; VTE; pneumonia; LOS
Vasileiou *et al.*, 2020 ^13^	Prospective cohort	6/35	29 ± 7	Not specified	9 (25%)	3 ^rd^ generation cephalosporin, fluoroquinolene, clindamycin, aminoglicoside, sulfonamide, piperacillin/tazobactam, amoxicillin clavulanate, metronidazole	Not specified	Complication; LOS
Yang *et al.*, 2021 ^14^	Retrospective cohort	2996/7275	<20: 15%; 20–24: 21.8%; 25–29: 21.1%; 30–34: 17.6%; 35–49: 24.4%	Not specified	Not specified	Not specified	Open and laparoscopic	Fetal loss; mode of delivery; preterm delivery

^*^ Mean.

^†^ Median.

We used the ROBINS-I tool to assess the risk of bias, and the results are presented in Table 2. We judged the study by Nakashima *et al*. to have a serious risk of bias because there was a significantly higher rate of complicated appendicitis in the surgery group. We assessed the remaining study to be of moderate risk of bias because confounding had been sufficiently controlled. However, the possibility of unmeasured confounding variables inherent in the design of non-randomized studies gave a moderate risk of confounding bias. We judged bias from the outcome measurement to be low because although no blinding was used, the outcomes were fairly objective. We did not perform analysis for publication bias because funnel plot analysis or other statistical methods did not have enough power to detect bias in a small number of studies.

**Table 2.** Risk of bias assessment The Risk of Bias in Non-Randomized Studies – of Interventions (ROBINS-I) tool.

Authors	Confounding	Selection	Intervention classification	Deviation from intervention	Missing data	Measurement of outcome	Selection of reported results	Overall
Abbasi *et al.*, 2014 ^1^	Moderate	Low	Low	Low	Low	Low	Low	Moderate
Liu *et al.*, 2021 ^6^	Moderate	Low	Low	Low	Low	Low	Low	Moderate
Nakashima *et al.*, 2021 ^7^	Serious	Low	Low	Low	Low	Low	Low	Serious
Vasileiou *et al.*, 2020 ^13^	Moderate	Low	Low	Low	Low	Low	Low	Moderate
Yang *et al.*, 2021 ^14^	Moderate	Low	Low	Low	Low	Low	Low	Moderate

### Preterm delivery

**Figure 2.**
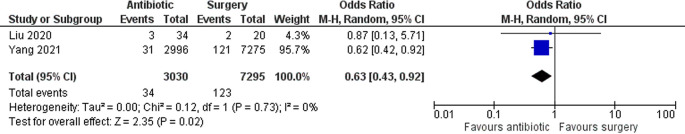
Forest plot showing preterm delivery between antibiotic and surgery group.

Two studies with a total of 10,325 patients assessed the occurrence of preterm labor. Overall, patients in the antibiotic group had a significantly lower risk of preterm delivery (OR 0.63 [95% CI 0.43–0.92]; p 0.02) ( Figure 2).

### Fetal loss

**Figure 3.**
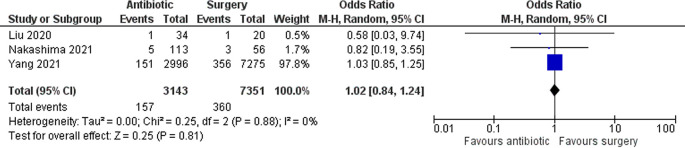
Forest plot showing fetal loss between antibiotic and surgery group.

Three studies with a total of 10,494 patients assessed the occurrence of fetal loss. Overall, there were no significant differences in fetal loss between the two groups (OR 1.02 [95% CI 0.84–1.24]; p 0.81) ( Figure 3).

### Maternal death

From the two studies with a total of 223 patients, there was no report of maternal death either in the antibiotic or the surgery group. Therefore, a meta-analysis could not be performed.

### Complications

**Figure 4.**
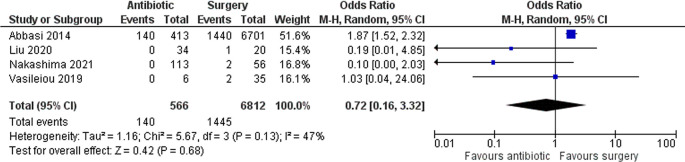
Forest plot showing complications between antibiotic and surgery group.

Four studies with a total of 7,378 patients assessed complications. Abbasi *et al*. included sepsis, septic shock, peritonitis, and VTE as complications. Liu *et al*. reported one grid iron complication in the surgery group. Nakashima *et al*. included surgical site infection, VTE, and pneumonia as complications. Lastly, Vasileiou *et al*. used Clavien-Dindo complications. Overall, there was a lower risk of complications in the antibiotic group, but it was not statistically significant (OR 0.72 [95% CI 0.16–3.32]; p 0.68) ( Figure 4). However, only Abbasi *et al*. reported peritonitis, and they found that patients in the antibiotic group significantly had a higher rate of peritonitis (28.8% vs. 19.8%; p < 0.001).

### Sensitivity analysis

**Figure 5.**
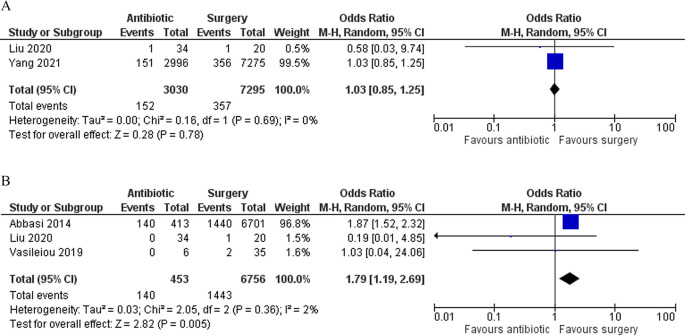
Forest plot showing the sensitivity analysis for fetal loss (A) and complications (B) between antibiotic and surgery group.

We excluded the study by Nakashima *et al*. in our sensitivity analysis. Regarding fetal loss, the result did not differ from our primary analysis. However, regarding the outcome, we found that patients in the antibiotic group significantly had a higher rate of complications (OR 1.79 [95% CI 1.19–2.69]; p 0.005) ( Figure 5).

### Certainty of evidence

The certainty of the evidence was very low to low ( Table 3). Evidence regarding fetal loss and complication had a serious risk of bias and inconsistency, so we judged them to be of very low quality. Evidence regarding fetal loss did not have serious concerns, but we assessed them to be of low quality due to the observational design of studies. Table 3 gives a summary of the assessment.

**Table 3.** Certainty of evidence.

Outcome	No. of studies	Study design	Risk of bias	Inconsistency	Indirectness	Imprecision	Other consideration	OR (95% CI)	Overall
Preterm delivery	3	Observational	Serious ^*^	Not serious	Not serious	Serious ^†^	Plausible residual confounding, would suggest spurious effect	1.02 (0.84 – 1.24)	Very low
Fetal loss	2	Observational	Not serious	Not serious	Not serious	Not serious	None	0.63 (0.43 – 0.92)	Low
Complication	4	Observational	Serious ^*^	Serious ^‡^	Not serious	Serious ^†^	Plausible residual confounding, would suggest spurious effect	0.72 (0.16 – 3.32)	Very low

^*^ One study rated as having a serious risk of bias. Downgraded by one point.

^†^ 95% CI overlaps no effect. Downgraded by one point.

^‡^ *I* ^2^ > 40%. Downgraded by one point.

## Discussion

Our analysis showed that conservative management of acute appendicitis in pregnant patients resulted in a lower risk of preterm labor (OR 0.63 [95% CI 0.43–0.92]; p 0.02). The rate of complications in our primary analysis was also comparable between the two groups. However, after excluding the study by Nakashima *et al*. in our sensitivity analysis, we found that antibiotic treatment resulted in a significantly higher risk of complications (OR 1.79 [95% CI 1.19–2.69]; p 0.005). The result of this analysis mainly came from the study by Abbasi *et al*., which had the highest number of participants. Abbasi *et al*. was also the only one that reported the occurrence of sepsis and peritonitis. ^12^ Several newer studies have suggested that conservative management is a safe option in treating acute appendicitis in the pregnant population. ^6^^,^^7^^,^^13^ Interestingly, none of those studies found any occurrence of sepsis and peritonitis. We suspected those studies did not have adequate statistical power to detect those complications. Immunological changes during pregnancy are one of the possible reasons for an increase in infectious complication, such as peritonitis and sepsis. Immune response in patients with acute appendicitis includes increased neutrophil and C-reactive Protein (CRP). ^15^ During pregnancy, there is evidence of increased neutrophil activation, but its activity and phagocytosis function are limited. ^16^ In conclusion, decreased activities of activated neutrophils in pregnancy may explain higher rates of infectious complications, such as sepsis, in the pregnant population.

Another concern is the difference in the number of patients with complicated appendicitis. Abbasi *et al*. in their study, had 20.3% of the study population diagnosed with complicated appendicitis, ^12^ Vasileiou *et al*. had 25%, ^13^ while Liu *et al*. only included patients with uncomplicated appendicitis. ^6^ Liu *et al*. in their study, expectedly did not report a higher complication rate in the antibiotic group. ^6^ However, Vasileiou *et al*., comparable with the study by Abbasi *et al*. in terms of patients with complicated appendicitis also reported a lower rate of complication. ^13^ This made it difficult to arrive at a definite conclusion on whether patients with complicated appendicitis could benefit from conservative management.

Despite a higher risk of complications, we found that conservative management reduced the risk of preterm labor. Therefore, the potential benefits of conservative management should not be crossed out just yet. Two meta-analyses on non-pregnant patients also concluded that conservative management is a viable option. ^10^^,^^11^ Aside from the studies included in the analysis, there were two studies that also supported the use of antibiotic for acute appendicitis in the pregnant population. However, we excluded those two studies because they did not provide a comparison with a control group. ^5^^,^^17^ Rather than focusing solely on comparing the efficacy and safety between conservative and surgical approaches, more attention should be given to tailoring a strategy for each patient. Several studies have identified predictors for the failure of conservative management in non-pregnant patients, such as high serum C-reactive protein, presence of appendicolith, diabetes, longer duration of symptoms, higher temperature, higher Alvarado score, and larger appendiceal diameter. ^18^^–^^20^ However, those studies only studied patients with uncomplicated appendicitis.

Diagnosis of acute appendicitis in pregnancy also has its consideration. Negative appendectomy in the pregnant population ranges from 25 to 50%, higher than those in the general population, which only ranges from 15 to 35%. ^21^ To reduce unneeded surgery, radiographical imaging is usually ordered to supplement the diagnosis. As computed tomography (CT) scan is often contraindicated in pregnant patients, ultrasonography and magnetic resonance imaging (MRI) are the main radiographical modalities for diagnosing appendicitis. As ultrasonography has a low sensitivity and specificity, MRI is often warranted in patients with a negative ultrasonography finding. ^22^^,^^23^ However, MRI is not widely available, especially in low to middle-income settings. Therefore, in those settings, it can be expected that a significant number of pregnant patients with suspected appendicitis would receive a misdiagnosis. In a pregnant patient with a doubtful clinical presentation of acute appendicitis and a negative ultrasonography finding, a conservative approach might be appropriate as first-line management. However, a surgical approach should always be preferred for a patient with a set of specific and characteristic symptoms of acute appendicitis.

We listed comparison as surgical treatment, either open or laparoscopic based on insufficient evidence of suspected association between laparoscopic appendectomy and miscarriage. This association needs further research which results in no recommendation of one method superior of the other. Furthermore, resources needed to carry out laparoscopic procedures in developing countries are still limited - hence open appendectomy remains a viable and equal option. ^23^

One limitation of the available studies on this subject is the lack of information on whether appendicitis recurred or not after conservative management. Vasileiou *et al*. reported that patients in the antibiotic and surgery group had a comparable emergency department (ED) return rate after 30 days. ^13^ However, they did not specify the reason for the ED visit. One meta-analysis by Sallinen *et al*. found that 8.5% of patients in the antibiotic group required an appendectomy within one month. ^10^ Future studies intending to study conservative management for acute appendicitis in pregnant patients should include recurrence as one of the outcomes.

Additionally, the use of tocolytic agents was not mentioned in any of the journals included in this review. Although there had been some evidence in the past regarding the function of tocolytic agents administration for pregnant women undergoing surgery, latest systematic review and recommendation do not recommend its use because it has not been shown to improve outcomes. ^24^ Lastly, the studies did not provide information regarding complications in accordance with each trimester. There has been evidence in the past regarding lower risk of acute appendicitis in the third trimester of pregnancy, therefore, future studies should evaluate any complication in accordance with the patients’ trimester. ^25^

Our analysis has several limitations. One limitation is the small number of studies and the non-randomized design. It is indeed difficult to perform a clinical trial on pregnant patients. Therefore, future cohorts should be carefully designed as they are the main modality to address this issue. Another limitation is the potential risk of confounding bias. We addressed this issue by performing a sensitivity analysis. However, unmeasured confounding inherent in the design of cohort studies remained a problem.

## Conclusion

Patients in the antibiotic group had a significantly lower risk of preterm labor but a higher risk of complications. Seeing the result and the available evidence, we suggested that conservative management should not be given indiscriminately to pregnant patients until it is found which patients could benefit the most from such a strategy. Conservative management must also be given with close and routine monitoring to prevent life-threatening complications, such as sepsis and peritonitis. Nevertheless, the potential benefit should warrant future studies addressing the identified concerns, namely patient selection and recurrence.

## Data Availability

All data underlying the results are available as part of the article and no additional source data are required. Zenodo: Antibiotic versus surgery in the treatment of acute appendicitis in the pregnant population: A systematic review and meta-analysis, https://doi.org/10.5281/zenodo.7538790. ^26^ This project contains the following extended data:
- Database and Literature Search - Fig 1. PRISMA flow diagram. - Fig 2. Forest plot showing preterm delivery between antibiotic and surgery group. - Fig 3. Forest plot showing fetal loss between antibiotic and surgery group. - Fig 4. Forest plot showing complications between antibiotic and surgery group. - Fig 5. Forest plot showing the sensitivity analysis for fetal loss (A) and complications (B) between antibiotic and surgery group. -
Table 1. Characteristics of Included Studies. -
Table 2. Risk of bias assessment The Risk of Bias in Non-Randomized Studies – of Interventions (ROBINS-I) tool. -
Table 3. Certainty of evidence. Zenodo: PRISMA checklist for ‘Antibiotic versus surgery in the treatment of acute appendicitis in the pregnant population: A systematic review and meta-analysis’, https://doi.org/10.5281/zenodo.7538790. ^26^ Data are available under the terms of the Creative Commons Attribution 4.0 International license (CC-BY 4.0).
