# Supplementary material for: Antibiotic versus surgery in the treatment of acute appendicitis in the pregnant population: A systematic review and meta-analysis
Source: F1000Res. 2023 Sep 6;12:188. Originally published 2023 Feb 16. [Version 2] doi: 10.12688/f1000research.129906.2 (PMC11196932; doi:10.12688/f1000research.129906.2)
Supplement: Peer review history

# Reviewer report: Reviewer response for version 2

**DOI:** 10.5256/f1000research.155547.r294632

**Published:** 2024-07-08

**Authors:** Goran Augustin

**Affiliations:** 1 University of Zagreb, Zagreb, Croatia

A small number of studies were included. All were retrospective and non-randomized. Difficult to compare. it is unexpected that antibiotic treatment results in more complications. This bias is due to a population-based study of Abbas with more than 7000 people. this could lead to wrong conclusions. The same issue was with comparing open and laparoscopic appendectomy and outcome when the study by McGory (2007) was also nationwide database driven. Such large databases carry a big potential for errors. It was nicely uncovered in the article Augustin G, et al.,2020 [Ref-1].

Are the rationale for, and objectives of, the Systematic Review clearly stated?

Yes

Is the statistical analysis and its interpretation appropriate?

I cannot comment. A qualified statistician is required.

If this is a Living Systematic Review, is the ‘living’ method appropriate and is the search schedule clearly defined and justified? (‘Living Systematic Review’ or a variation of this term should be included in the title.)

Not applicable

Are sufficient details of the methods and analysis provided to allow replication by others?

Yes

Are the conclusions drawn adequately supported by the results presented in the review?

Yes

Reviewer Expertise:

Abdominal surgery

I confirm that I have read this submission and believe that I have an appropriate level of expertise to confirm that it is of an acceptable scientific standard, however I have significant reservations, as outlined above.

## References

[1] : Discordant outcomes of laparoscopic versus open appendectomy for suspected appendicitis during pregnancy in published meta-analyses: an overview of systematic reviews. *Surg Endosc* .2020;34(10) : 10.1007/s00464-020-07674-6 4245-4256 10.1007/s00464-020-07674-6 32556754

# Reviewer report: Reviewer response for version 2

**DOI:** 10.5256/f1000research.155547.r294634

**Published:** 2024-06-24

**Authors:** kumar Hari Rajah

**Affiliations:** 1 Taylors University School of Medicine and Health Science, Selangor, Malaysia

This systemic review and meta-analysis on antibiotic versus surgery in the management of acute appendicitis in pregnancy. I would suggest adding laparoscopic appendectomy and open appendectomy to the keywords. The PRISMA guide is done properly and the studies and type are clearly stated .The results section is clearly defined and the discussion and conclusions are consistent with the evidence provided.  The references are adequate.

Are the rationale for, and objectives of, the Systematic Review clearly stated?

Yes

Is the statistical analysis and its interpretation appropriate?

Yes

If this is a Living Systematic Review, is the ‘living’ method appropriate and is the search schedule clearly defined and justified? (‘Living Systematic Review’ or a variation of this term should be included in the title.)

Yes

Are sufficient details of the methods and analysis provided to allow replication by others?

Yes

Are the conclusions drawn adequately supported by the results presented in the review?

Yes

Reviewer Expertise:

general surgery and gastroenterology

I confirm that I have read this submission and believe that I have an appropriate level of expertise to confirm that it is of an acceptable scientific standard.

# Reviewer report: Reviewer response for version 2

**DOI:** 10.5256/f1000research.155547.r204328

**Published:** 2023-09-26

**Authors:** Rıfat Peksöz

**Affiliations:** 1 Department of General Surgery, Faculty of Medicine, Ataturk University, Erzurum, Erzurum, Turkey

Necessary revisions have been made. I wish success to the authors.

Are the rationale for, and objectives of, the Systematic Review clearly stated?

Partly

Is the statistical analysis and its interpretation appropriate?

Yes

Are sufficient details of the methods and analysis provided to allow replication by others?

Yes

Are the conclusions drawn adequately supported by the results presented in the review?

Yes

Reviewer Expertise:

My areas of expertise are general surgery, especially acute appendicitis. Surgery interest: (General surgery, Gastrointestinal diseases): Emergencies in general surgery, ERCP, Endoscopy-colonoscopy, endoscopic interventions, colo-rectal diseases (colorectal cancers, diverticülitis), stomach diseaes (gastric cancer), gastrointestinal cancers, open or laparoscopic hernias (groin hernias, incisional, umbilical), anorectal diseases (pilonidal sinüs, hemorrhoid, anal fissure, perianal fistüla...), gallbladder surgery, spleen surgery, bariatric surgery (morbid obesity), nissen fundoplication.

I confirm that I have read this submission and believe that I have an appropriate level of expertise to confirm that it is of an acceptable scientific standard.

# Reviewer report: Reviewer response for version 1

**DOI:** 10.5256/f1000research.142624.r195177

**Published:** 2023-09-01

**Authors:** Rıfat Peksöz

**Affiliations:** 1 Department of General Surgery, Faculty of Medicine, Ataturk University, Erzurum, Erzurum, Turkey

The authors aimed to investigate the current review to identify the efficacy and safety of conservative treatment in the treatment of acute appendicitis in pregnancy in this study.

 Acute appendicitis is the most frequently seen surgical emergency in pregnant women and the most frequently encountered cause of non-obstetric acute abdomen. Due to the physiological and anatomical changes that occur during pregnancy and the limited use of radiological methods, it is difficult to diagnose AA during pregnancy. These conditions increase the risk of morbidity and mortality. Studies on acute appendicitis in pregnant women will provide a better understanding of this issue. In this regard, this study is very important in terms of contributing to the literature. Howewer, some issues should be raised from my point of view. I hope my comments contribute to your article, good luck to the authors.

 Plagiarism rate: didn’t evaluate because of this paper’s pre-review form published on the F1000 research page.

Comments to the author(s):

1. In the Abstract section, the complications which are seen more in the results section should be mentioned a little bit. This can be difficult to do, but when researchers read the articles, they read the abstract first. It is important to provide this information in order for the study to be more effective.
2. Keywords should be rewritten, comments; Add 'antibiotic therapy' instead of 'Antibiotic'. Add 'Pregnancy' instead of 'pregnant', and, add outcomes or complications words.
3. In your article, it was emphasized that the treatment of acute appendicitis in pregnant women is controversial, but in the **introduction section *,*** only the superiority of antibiotic therapy was emphasized, and the study highlighting surgery was not emphasized. I recommend that this study with good surgical results be emphasized in this section.
4. Discussion section *: ‘’Immunological changes during pregnancy are one of the possible reasons for an increase in infectious complication, such as peritonitis and sepsis. Immune response in patients with acute appendicitis includes increased neutrophil and C-reactive Protein (CRP)’’. ^(^* ^15, doi: 10.1111/ans.17443)^
  I suggest adding an additional reference at the end of this sentence. Our work on this subject is up-to-date and has received many references in a short time (Peksöz *et al.*, 2022 ^1^).
5. Discussion section *:* *"We listed comparison as surgical treatment, either open or laparoscopic based on insufficient evidence of suspected association between laparoscopic appendectomy and miscarriage. This association needs further research which results in no recommendation of one method superior of the other. Furthermore, resources needed to carry out laparoscopic procedures in developing countries are still limited - hence open appendectomy remains a viable and equal option. ^23"^*
6. Disadvantages of LA were mentioned in your article, but Laparascopic appendectomy has been routinely applied in pregnant women today. Although the subject of your study is antibiotic treatment or surgical treatment, this current information should be renewed and reviewed. An important study demonstrated the superiority of LA. ‘’Compared with OA, LA was associated with lower wound infection rates and shorter LOS. Based on our results and recent literatures, we suggest that LA shows non-inferior safety with respect to pregnancy outcomes but superior with regard to surgical outcomes compared with OA in pregnant women with suspected appendicitis’’ (Lee *et al.*, 2019 ^2^).

**Evaluation report:** Congratulations to the authors for good work, this study well written. Introduction, materials and methods, results and discussion sections of the article have enough scientific knowledge. I think it is acceptable for indexing after minör revisions.

Are the rationale for, and objectives of, the Systematic Review clearly stated?

Partly

Is the statistical analysis and its interpretation appropriate?

Yes

Are sufficient details of the methods and analysis provided to allow replication by others?

Yes

Are the conclusions drawn adequately supported by the results presented in the review?

Yes

Reviewer Expertise:

My areas of expertise are general surgery, especially acute appendicitis. Surgery interest: (General surgery, Gastrointestinal diseases): Emergencies in general surgery, ERCP, Endoscopy-colonoscopy, endoscopic interventions, colo-rectal diseases (colorectal cancers, diverticülitis), stomach diseaes (gastric cancer), gastrointestinal cancers, open or laparoscopic hernias (groin hernias, incisional, umbilical), anorectal diseases (pilonidal sinüs, hemorrhoid, anal fissure, perianal fistüla...), gallbladder surgery, spleen surgery, bariatric surgery (morbid obesity), nissen fundoplication.

I confirm that I have read this submission and believe that I have an appropriate level of expertise to confirm that it is of an acceptable scientific standard, however I have significant reservations, as outlined above.

## References

[1] : Significance of laboratory parameters in diagnosing acute appendicitis during pregnancy. *ANZ J Surg* .2022;92(1-2) : 10.1111/ans.17443 121-127 10.1111/ans.17443 34967103

[2] : Laparoscopic appendectomy versus open appendectomy for suspected appendicitis during pregnancy: a systematic review and updated meta-analysis. *BMC Surg* .2019;19(1) : 10.1186/s12893-019-0505-9 41 10.1186/s12893-019-0505-9 31023289 PMC6482586

# Response

**Published:** 2023-09-02

**Authors:** Valeska Candrawinata

**Affiliations:** National Institute of Health Research and Development, Indonesia, Indonesia

Dear Reviewer, we thank you for your kind review and insightful notes. We have revised the manuscript accordingly.

1. In the Abstract section, the complications which are seen more in the results section should be mentioned a little bit. This can be difficult to do, but when researchers read the articles, they read the abstract first. It is important to provide this information in order for the study to be more effective.

**Response**: We have revised the abstract accordingly and added more information about the complications in the abstract.

1. Keywords should be rewritten, comments; Add 'antibiotic therapy' instead of 'Antibiotic'. Add 'Pregnancy' instead of 'pregnant', and, add outcomes or complications words.

**Response**: We have revised the keywords accordingly.

  

1. In your article, it was emphasized that the treatment of acute appendicitis in pregnant women is controversial, but in the **introduction section *,*** only the superiority of antibiotic therapy was emphasized, and the study highlighting surgery was not emphasized. I recommend that this study with good surgical results be emphasized in this section.

**Response**: We have revised this section accordingly and added them.

  

1. Discussion section *: ‘’Immunological changes during pregnancy are one of the possible reasons for an increase in infectious complication, such as peritonitis and sepsis. Immune response in patients with acute appendicitis includes increased neutrophil and C-reactive Protein (CRP)’’.* *^(^* ^15, doi: 10.1111/ans.17443)^
  I suggest adding an additional reference at the end of this sentence. Our work on this subject is up-to-date and has received many references in a short time (Peksöz *et al.*, 2022 ^1^ ).

**Response**: We have added the reference.

  

1. Discussion section *:* *"We listed comparison as surgical treatment, either open or laparoscopic based on insufficient evidence of suspected association between laparoscopic appendectomy and miscarriage. This association needs further research which results in no recommendation of one method superior of the other. Furthermore, resources needed to carry out laparoscopic procedures in developing countries are still limited - hence open appendectomy remains a viable and equal option.* *^23"^*

**Response**: We have revised the article accordingly.

  

1. Disadvantages of LA were mentioned in your article, but Laparascopic appendectomy has been routinely applied in pregnant women today. Although the subject of your study is antibiotic treatment or surgical treatment, this current information should be renewed and reviewed. An important study demonstrated the superiority of LA. ‘’Compared with OA, LA was associated with lower wound infection rates and shorter LOS. Based on our results and recent literatures, we suggest that LA shows non-inferior safety with respect to pregnancy outcomes but superior with regard to surgical outcomes compared with OA in pregnant women with suspected appendicitis’’ (Lee *et al.*, 2019 ^2^ ).

**Response**: We have revised the article accordingly.
